# A pediatric patient with bilateral tracheobronchial foreign body successfully treated using surgical intervention with extracorporeal circulation: case report and literature review

**DOI:** 10.3389/fped.2024.1406195

**Published:** 2024-06-12

**Authors:** Li-Qiang Xu, Qiang Liu, Min Zeng, Hui-Zhi Yan, Nan Hu, Qun-Xian Zhang, Qiang Guo, Jia-Long Guo, Jun Zhang

**Affiliations:** Department of Cardiothoracic Surgery, Taihe Hospital, Hubei University of Medical, Shiyan, China

**Keywords:** tracheobronchial foreign body, fiber bronchoscopy, extracorporeal circulation, literature review, CT

## Abstract

Unilateral Tracheobronchial foreign body (TFB) present a common clinical disease, whereas bilateral TFB is a rare and acute condition associated with high mortality rates. This case study discusses a pediatric patient hospitalized due to respiratory distress following accidental ingestion of peanut kernels. A plain chest CT scan revealed obstructive emphysema in the right main bronchus and a foreign body at the opening of the left main bronchus. Surgical removal of the bilateral TFB under extracorporeal circulation resulted in a successful postoperative recovery, leading to discharge on the 9th day. A comprehensive literature search was conducted across databases including PubMed, Web of Science, EMBASE, Cochrane Library, and CNKI, spanning publications from January 2014 to October 2023, utilizing keywords “bronchial foreign body” and “Peanut”. After deduplication and relevance screening, 9 pertinent literature sources were included. The objective of this study is to enhance clinical practitioners' understanding of TFB management and improve diagnostic and treatment capabilities through analysis of age of onset, clinical manifestations, diagnosis, and treatment approaches in critically ill pediatric patients.

## Introduction

Tracheobronchial foreign body (TFB) are a common clinical condition with a high incidence rate. Symptoms of TFB in children may manifest as cough, wheezing, and fever. Severe cases with severe cases potentially causing breathing difficulties, suffocation, and life-threatening situations. This condition is often an acute and severe illness encountered in children under 3 years of age. Prompt diagnosis and timely removal of TFB are critical for clinical management. Delayed or inadequate diagnosis and treatment may lead to fatal outcomes for the child.

**Figure 1:**
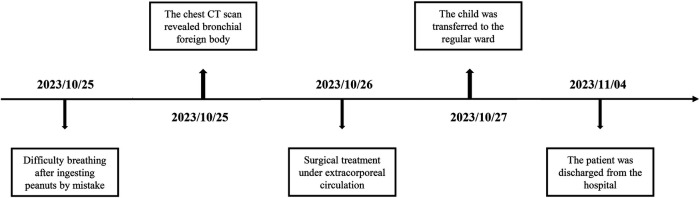
Timeline of diagnosis, and treatment in this case. CT, computed tomography.

In recent years, fiberoptic bronchoscopy, due to continuous advancements in medical technology, has become a prevalent modality for diagnosing and managing TFB in children. It is considered a preferred method for diagnosing and treating TFB in child and has emerged as the primary technique for their removal (1). However, this study reported a rare case of a child with a TFB using surgery. However, this study presents a rare case of a child with a TFB necessitating surgical intervention. The child, experiencing severe respiratory distress, exhibited bilateral TFB obstruction on chest CT scan, and bilateral TFB were removed using surgical treatment with the support of extracorporeal circulation technology. The patient showed significant postoperative recovery (Figure 1). Additionally, we conducted a comprehensive literature search across PubMed, Web of Science, EMBASE, Cochrane Library, and CNKI databases to examine the age of onset, diagnosis, and treatment of TFB in child, with the aim of enhancing understanding in the diagnosis and management of this condition.

## Case description

In October 2023, a 1-year-old child from Shiyan city in China ingested peanuts mistakenly, leading to crying and a paroxysmal, irritating cough, accompanied by difficulty breathing for 4 h. Upon admission, the patient presented with non-projectile vomiting of gastric contents and gradual lip cyanosis. Physical examination findings included a temperature of 37.3 °C, heart rate of 187 beats/min, respiratory rate of 69 breaths/min, and blood pressure of 90/47 mmHg. The child appeared agitated and teary, showing signs of dyspnea, visible retractions during breathing, a pallid complexion, and cyanosis of the lips. Reduced breath sounds and audible wheezing were present in both lungs, along with a faint rale in the lower left lung. Cardiac examination revealed a regular rhythm, strong heart sounds, and the absence of pathological murmurs.

**Figure 2:**
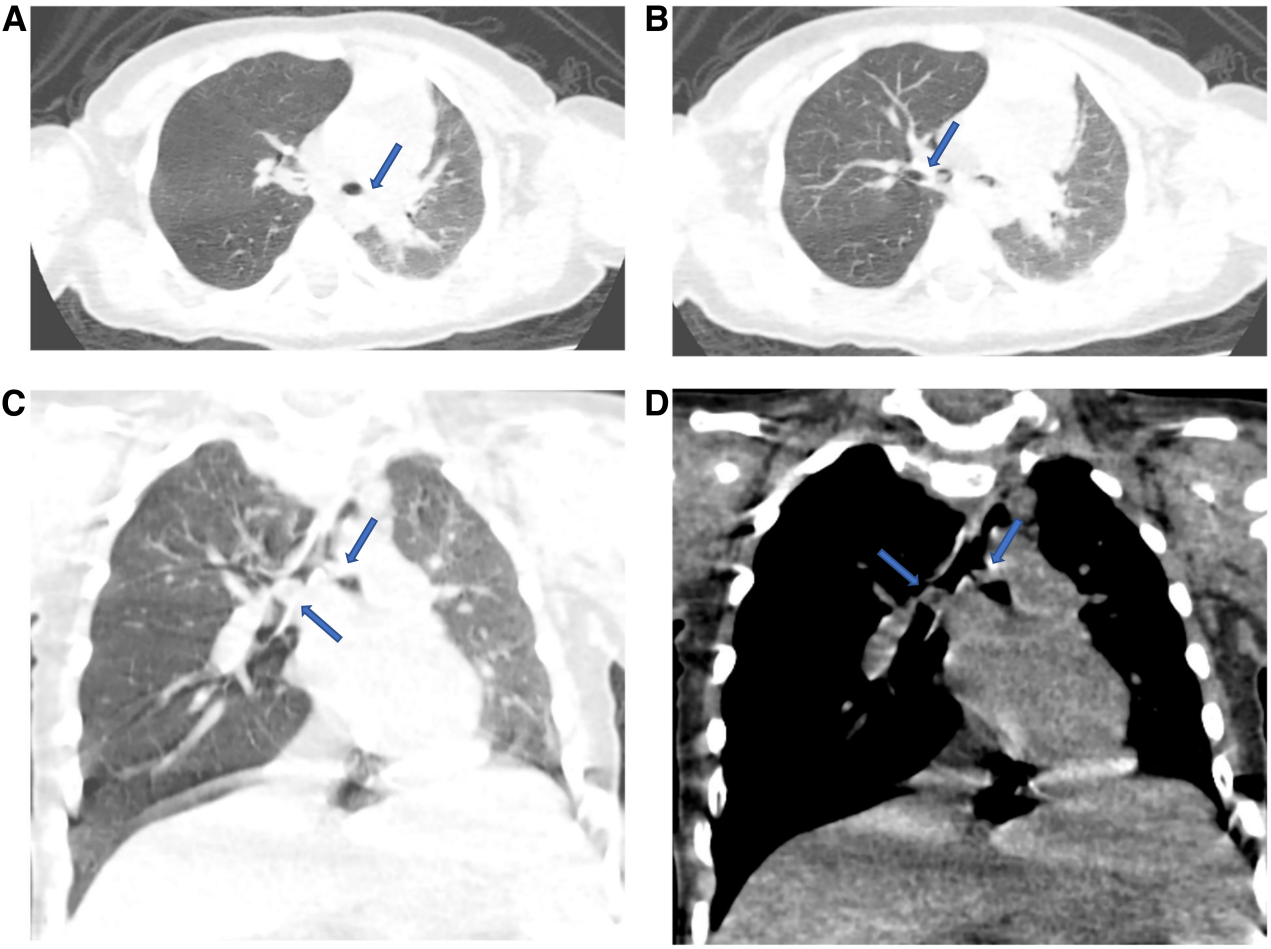
The location of bilateral TFB in patient were showed using the CT plain scan. (**A,B**) Bilateral TFB were shown from pulmonary window; (**C,D**) bilateral TFB were shown after three-dimensional reconstruction. TFB, tracheobronchial foreign body.

The laboratory tests did not reveal any significant abnormalities. The electrocardiogram indicated sinus tachycardia and incomplete right bundle branch block. Chest CT scan findings showed a 0.6 × 0.3 cm nodular high-density shadow in the child's right main bronchus, accompanied by increased volume and reduced density in the right lung. Additionally, a slight leftward displacement of the mediastinum was noted. A strip-shaped translucent shadow was observed at the apex of the right lung, along with a suspicious high-density shadow at the opening of the left main bronchus (Figure 2). The child was diagnosed with critical TFB. The presence of respiratory distress and cyanosis indicated obstruction from bilateral TFB. Given the risk of airway obstruction due to peanut fragmentation during bronchial procedures, surgical intervention offered comprehensive bronchial visualization, ensuring precise and clear surgical outcomes. Consequently, emergency surgery was conducted with extracorporeal circulation from the right atrium to the ascending aorta for bilateral TFB removal.

### Surgical procedure

**Figure 3:**
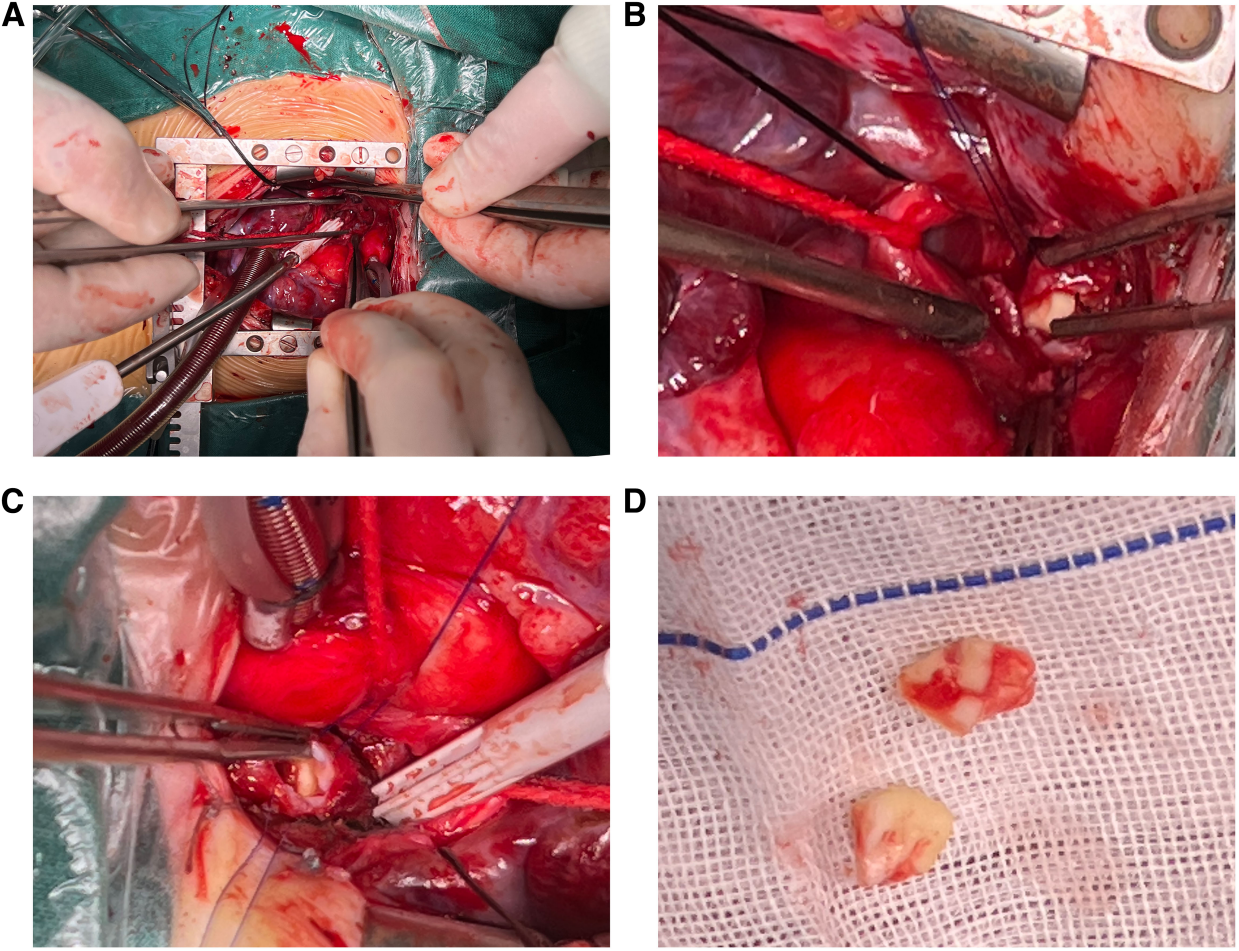
The bilateral TFB were removed through surgical procedures under extracorporeal circulation. (**A**) Extracorporeal circulation; (**B,C**) visualization of Bilateral TFB; (**D**) bilateral TFB. TFB, tracheobronchial foreign body.

The child is positioned supine, and arterial and venous monitoring is initiated. Standard disinfection and draping protocols are adhered to. A midline incision is made in the chest, followed by sternotomy to access the heart. Whole-body heparinization is administered. Right atrial blood drainage is performed through a catheter at the right atrial appendage, and a blood supply catheter is inserted via the ascending aorta to establish extracorporeal circulation (Figure 3). To fully expose the trachea, carina, and left and right main bronchi, gentle retraction of the aorta, superior vena cava, and main trunk of the right pulmonary artery is conducted. A longitudinal incision and gentle retraction of the right main bronchus reveal a peanut kernel obstructing half of the lumen. The foreign body is extracted, and the bronchus is sutured with proline suture material (Figure 3). Similarly, the left main bronchus is incised to reveal an obstruction by half a peanut kernel. Following foreign body removal, the bronchus is sutured (Figure 3). Extracorporeal circulation is terminated, protamine is administered, hemostasis is achieved, the sternum is stabilized, and the incision is sutured.

**Figure 4:**
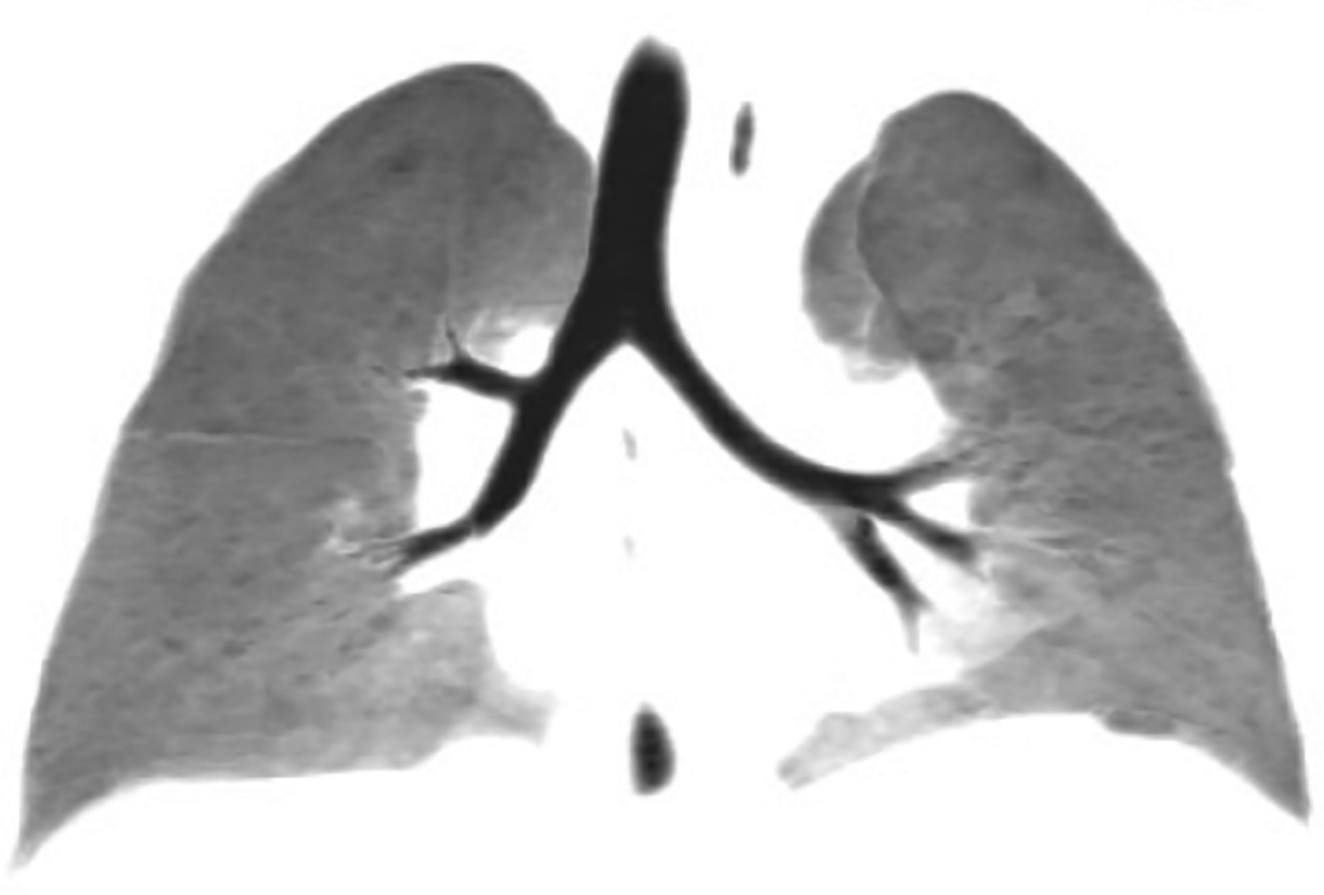
CT plain scan showed bilateral trachea patency and no obvious foreign bodies on the 9th day after surgery.

The child was transferred to a general ward the day after the surgery. A plain chest CT scan showed no signs of anastomotic stenosis or foreign bodies in either bronchus (Figure 4). Furthermore, fiberoptic bronchoscopy did not reveal any bronchial stenosis. The patient was discharged in good health on the 9th day post-surgery (Figure 1). Presently, the child does not experience any discomfort such as palpitations, chest tightness, or dyspnea.

## Literature review

The search encompassed PubMed, Web of Science, EMBASE, Cochrane Library, and CNKI databases. Keywords “tracheobronchial foreign body” and “Peanut” were used to identify relevant literature published between January 2014 and November 2023. After deduplication and exclusion of incomplete full-text submissions, and following the integration of clinical symptoms and imaging data, a total of 9 articles were selected. These articles encompassed 529 patients from external sources and 1 patient from our hospital, leading to a collective cohort of 530 patients (Table 1).

**Table 1:** Basic information included in the study.

Study	Year	Country	*N*	Therapeutic method	References
Ginter et al.	2023	United States	1	FB	(2)
Ünal et al.	2022	Turkey	20	FB	(3)
Salamah et al.	2022	Saudi Arabia	1	FB	(4)
Rance et al.	2022	France	12	FB	(5)
Wadhera et al.	2022	India	200	FB	(6)
Ding et al.	2020	China	200	FB or Surgery	(7)
Kim et al.	2018	Korea	20	FB	(8)
Azizoglu et al.	2016	Turkey	1	FB	(9)
Haddadi et al.	2015	Iran	74	FB	(10)

FB, fiber bronchoscopy.

**Figure 5:**
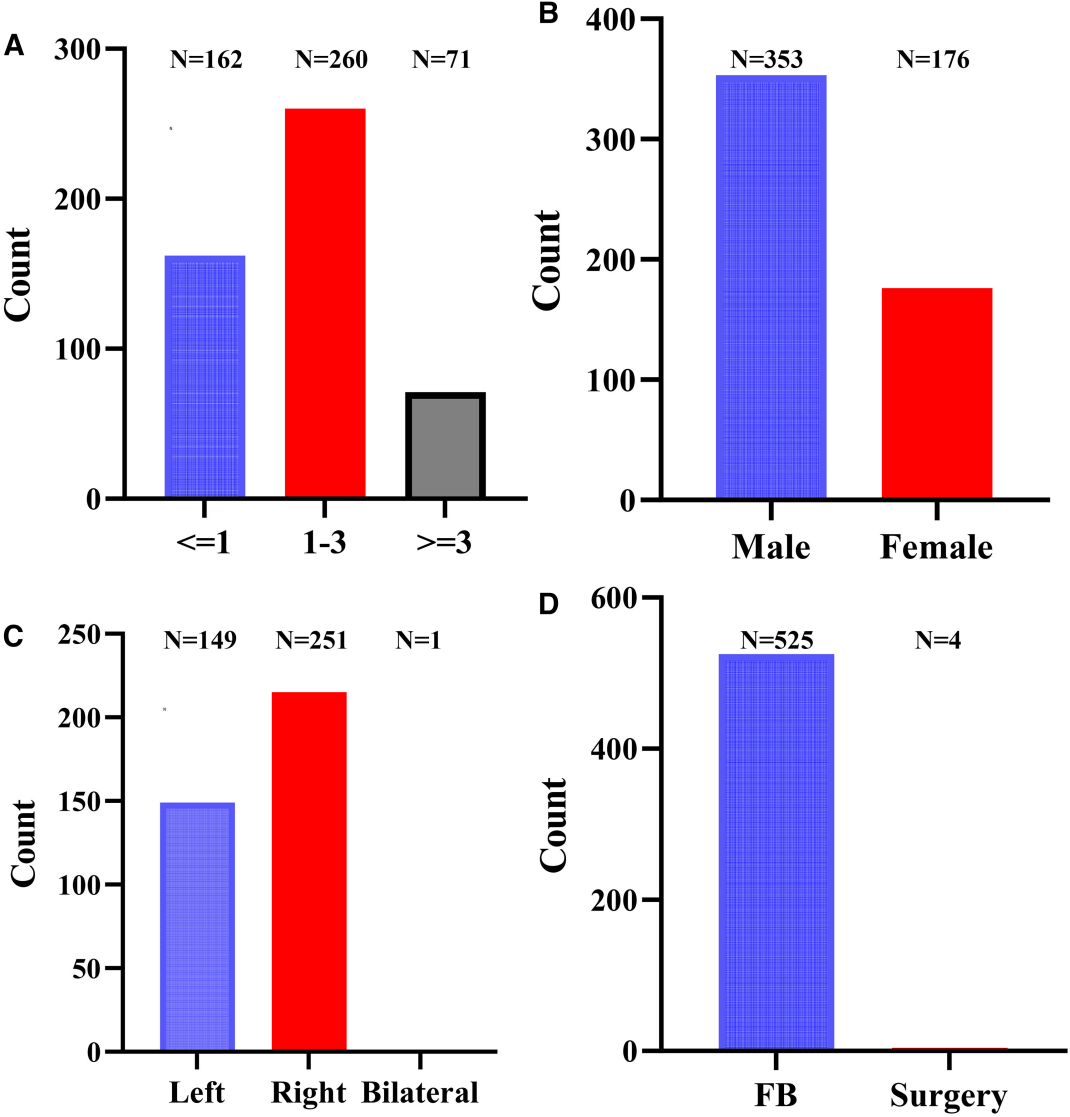
The clinical characteristics of literature review of patients. (**A**) Age; (**B**) sex; (**C**) foreign body location; (**D**) treatment of TFB. TFB, tracheobronchial foreign body; FB, fiber bronchoscopy.

The research findings are derived from diverse regions and countries, with peanuts being the predominant foreign body identified in the trachea and bronchi across 8 articles, accounting for 35.92% (190/529) of cases. These incidents primarily affect children under the age of 3, with the majority falling within the 1–3-year-old range (Figure 5A). Of the 529 patients, 353 were male (66.73%) and 176 were female (33.27%) (Figure 5B). The distribution of foreign bodies encompassed 149 cases in the left trachea and bronchi, 215 in the right, 31 in the main, and 1 in both trachea and bronchi (Figure 5C).

The main treatment modality for 529 children with TFB was fiberoptic bronchoscopy. More specifically, 524 children with unilateral TFB underwent fiberoptic bronchoscopy. One child with bilateral TFB was treated using fiberoptic bronchoscopy with veno-arterial extracorporeal membrane oxygenation (VA-ECMO) support, while 4 children with unilateral TFB required thoracotomy surgery due to insufficient efficacy of fiberoptic bronchoscopy treatment (Figure 5D).

## Discussion

The incidence of TFB is more prevalent in preschool children, particularly concentrated in infants and young children aged 1–3 years. The male-to-female ratio stands at approximately 2:1, potentially due to underdeveloped chewing and swallowing functions, alongside inadequate protective laryngeal reflexes in preschool-aged children. Furthermore, these children have immature teeth and may struggle to chew harder foods fully, increasing the risk of aspiration during crying or while eating. Children with TFB commonly exhibit symptoms like coughing, shortness of breath, and wheezing. In severe instances, pediatric patients may face breathing challenges and signs of suffocation, posing life-threatening risks. Some children may not display overt symptoms. TFB frequently occurs in the right bronchus, characterized by its relatively thick and short nature compared to the slender and lateral extension of the left bronchus. A study encompassing data from various regions identified peanuts as the predominant foreign body type, aligning with the 1–3 year age group. Of the patients, 353 (66.73%) were male and 176 (33.27%) were female, maintaining a male-to-female ratio close to 2:1. The study documented 149 cases of left TFB, 215 cases of right TFB, 31 cases of central TFB, and 1 case of bilateral TFB. Clinical manifestations among these patients included severe respiratory distress, cyanosis, critical conditions, and the urgent requirement for foreign body removal.

Bronchitis is characterized by inflammation of the bronchial mucosa, manifesting common symptoms such as cough, sputum production, and dyspnea. Similarly, bronchial tumors can present with comparable symptoms. Bronchial tuberculosis results from infection with Mycobacterium tuberculosis, leading to bronchial wall thickening, narrowing, and respiratory challenges. TFB may display symptoms akin to the aforementioned conditions, thus requiring careful differentiation. Now, the diagnostic and therapeutic strategies for TFB primarily encompass chest CT scans, bronchoscopy, and thoracotomy for foreign body retrieval. Currently, bronchoscopy plays a pivotal role in TFB management, allowing for direct and precise diagnostics. Fiberoptic bronchoscopy is extensively utilized due to its small diameter, flexibility, robust illumination, minimal invasiveness, straightforward operation, high safety profile, and clear visualization (11). The surgical success rate is notably high, offering confidence to the patients' families. Out of 529 children with TFB, 525 underwent fiberoptic bronchoscopy. Nevertheless, in select cases, open chest surgery was performed for successful foreign body removal, especially when bronchoscopy failed or in critical patient scenarios. Although unilateral TFB incidence is markedly prevalent globally across diverse age brackets, bilateral TFB occurrences in children are relatively infrequent. In this Chinese case, one bronchus is entirely obstructed, while the other is 70% blocked, resulting in asphyxiation in the child. Furthermore, the child's airways are susceptible to peanut fragmentation during bronchial procedures, increasing the likelihood of small airway obstructions. Surgical intervention offers a thorough assessment of both bronchi, guaranteeing precise and clear surgical procedures. Consequently, the child's parents are contemplating urgent surgical intervention with extracorporeal circulation.

Ginter et al., successfully removed bilateral TFB using fiberoptic bronchoscopy with VA-ECMO life support (2). Upon admission, the patient presented with cyanosis, respiratory distress, and a pallid complexion. Given the critical nature of the situation, emergency TFB surgery and life support therapy are imperative. Additionally, it is important to note that the use of neck blood vessels for VA-ECMO is both expensive and challenging for pediatric patients, such as the 1-year-old mentioned. Prolonged use of VA-ECMO post-surgery also poses thrombotic and other complications risks. Therefore, establishing extracorporeal circulation from the right atrium to the ascending aorta under open chest conditions is a more practical and cost-effective solution. This approach allows for swift withdrawal of extracorporeal circulation when promptly alleviating tracheal obstruction in pediatric patients. Fortunately, the patient was transferred to a regular ward on the first day after surgery and was discharged after recovery on the 9th day.

TFB incidents are particularly high in child aged 1–3 years old. If conditions permit, we also believe that fiberoptic bronchoscopy is the best way to remove bronchial foreign bodies. However, for child with unsatisfactory foreign body removal effects through fiberoptic bronchoscopy, open chest surgery may be selected to alleviate respiratory distress symptoms. In addition, it is also necessary to perform fiberoptic bronchoscopy after operation to understand bronchial stenosis. In summary, open chest surgery under cardiopulmonary bypass represents a safe and effective approach for treating critically ill child.

## Data Availability

The raw data supporting the conclusions of this article will be made available by the authors, without undue reservation.
